# Functional Features of Senescent Cells and Implications for Therapy

**DOI:** 10.3390/ijms26115390

**Published:** 2025-06-04

**Authors:** Tatiana V. Kirichenko, Yuliya V. Markina, Alexander M. Markin, Vyacheslav S. Vasilyev, Huiming Hua, Dahong Li, Anthony Yiu-Ho Woo, Roman V. Deev, Ilya I. Eremin, Konstantin V. Kotenko

**Affiliations:** 1 Petrovsky National Research Centre of Surgery, 119435 Moscow, Russia; t-gorchakova@mail.ru (T.V.K.); romdey@gmail.com (R.V.D.);; 2 Petrovsky Medical University, 119435 Moscow, Russia; 3 Medical Institute, Peoples’ Friendship University of Russia Named After Patrice Lumumba (RUDN University), 117198 Moscow, Russia; 4 Institute of Plastic Surgery and Cosmetology, 105066 Moscow, Russia; 5 Key Laboratory of Structure-Based Drug Design & Discovery of Ministry of Education, Shenyang Pharmaceutical University, Shenyang 110016, China; huimhua@163.com (H.H.);; 6 School of Traditional Chinese Materia Medica, Shenyang Pharmaceutical University, Shenyang 110016, China; 7 School of Life Sciences and Biopharmaceutics, Shenyang Pharmaceutical University, Shenyang 110016, China

**Keywords:** senescent cells, aging, senolytic therapy, SASP, mitochondrial dysfunction

## Abstract

Cellular senescence is a key mechanism of aging. Senescent cells negatively affect the function of tissues and organs, significantly contributimg to the aging of the organism. Functional and structural characteristics of senescent cells, such as genomic changes and cell cycle arrest, lysosome and mitochondrial dysfunction, and production of SASP factors, are promising therapeutic targets in the context of healthy longevity. The present review was designed to characterize the features of senescent cells in order to discuss current methods and problems of geroprotective therapy and perspective factors for the development of new strategies of anti-aging treatment. Publications were searched based on the analysis of articles containing the keywords “senescent cells, aging, senolytic therapy, SASP, mitochondrial dysfunction” in the PubMed and Scopus databases up to March 2025.

## 1. Introduction

Cellular senescence occurs at all stages of life and is an important physiological mechanism of tissue remodeling during embryogenesis, antitumor protection, and wound healing [1]. At the same time, increasing numbers of senescent cells in tissues is associated with aging of the organism, and senescence is also a pivotal determinant in the development and progression of chronic age-related diseases [2]. Macromolecular damage accumulating in senescent cells leads to dysfunction of organelles, disruption of the secretory activity of the cell with the development of the senescence-associated secretory phenotype (SASP), and structural changes in cells [3]. In turn, SASP factors induce the senescence of microenvironmental cells through paracrine and endocrine pathways [4]. Cellular senescence is characterized by cell cycle arrest due to replicative aging associated with telomere shortening, or DNA damage caused by various external and internal stress factors [5]. Understanding the molecular mechanisms underlying cellular senescence and identifying markers of senescent cells is important for developing target therapy aimed at eliminating senescent cells while preserving healthy, normally functioning cells.

Multiple experimental and clinical studies have been devoted to investigating the effect of senolytic therapy on the progression of age-related diseases and aging of the organism [6]. Geroprotective therapy strategies are based on the use of low-molecular-weight compounds that are cytotoxic to senescent cells, as well as on the use of drugs that modify the secretory phenotype of senescent cells [7]. However, the impact of longevity interventions on senescence burden has not been well-studied. Despite of the fact that preclinical studies of senolytic approaches have shown promising results, further research is needed to address problems such as side effects, duration of therapy, and indications for treatment. The present review was designed to characterize the features of senescent cells in order to discuss current methods and problems of geroprotective therapy as well as perspectives for the development of new strategies of anti-aging treatment. Publications were searched based on the analysis of articles containing the keywords “senescent cells, aging, senolytic therapy, SASP, mitochondrial dysfunction” in the PubMed and Scopus databases up to March 2025.

## 2. Markers of Senescent Cells Dysfunction

An increase in the number of senescent cells with age contributes to the development of chronic diseases; in particular, cardiovascular and degenerative diseases. Identification of senescent cells is important in basic and clinical research aimed at studying the pathogenesis of aging. Monitoring of senescent cell markers is used to develop diagnostic methods and approaches for geroprotective therapy [8]. However, a universal marker of cellular senescence is lacking due to a number of complexities associated with the phenotype heterogeneity and multiple physiological functions of senescent cells, which are especially important in the context of carcinogenesis, tissue regeneration, and embryonic development. The lack of a standardized method for determining cellular senescence makes it difficult to compare the results of different studies and hinders the implementation of basic research results into clinical practice [9]. To address these issues, current studies are devoted to identifying more specific biomarkers and developing new detection methods [10]. A common set of features is widely used to characterize cellular senescence. This includes assessment of the expression of cell cycle arrest markers, staining for senescence-associated β-galactosidase (SA-β-gal), and other markers described in this section.

### 2.1. Cell Cycle Arrest

Cellular senescence is accompanied by irreversible cell cycle arrest. There are two types of cellular aging: replicative senescence and stress-induced premature senescence (SIPS) [11]. Telomere shortening is the key marker of replicative aging. Telomeres are repeating nucleotide sequences at the ends of chromosomes that protect them from destruction or fusion with neighboring chromosomes. Telomere shortening occurs at a rate of 50–200 nucleotide pairs with each cell division until the number of cell divisions reaches the Hayflick limit due to critical shortening of telomeres, which causes cell cycle arrest [12]. The enzyme telomerase can synthesize terminal telomeric repeats using its own transferable RNA as a template, compensating for the decrease in telomere length during cell division [13]. Telomerase activity decreases with age and can be suppressed due to chronic inflammation, oxidative stress, and other pathological factors leading to telomere shortening and cellular senescence [14]. Telomere dysfunction caused by shortening and various factors causing DNA damage—in particular, oxidative stress, and the effects of oncogenes and environmental factors—leads to the development of DNA damage. Due to the DNA damage response (DDR), phosphorylation of histone H2AX and tumor suppressor p53 occurs, mediated by serine/threonine-specific protein kinases ATR and ATM [15]. Phosphorylation of histone H2AX is the earliest response to double-strand DNA breaks. The formation of γH2AX is a signal initiating DNA reparation. γH2AX has been identified as a marker of senescent neurons in old mice, as well as during embryogenesis [16]. Activation of p53 leads to cell cycle arrest by acting as a transcription factor that increases the expression of the cyclin-dependent kinase (CDK) inhibitor p21 [15]. CDKs have a key function in signaling pathways that regulate transcription and the cell cycle. The CDK inhibitors p21 and p16 block the phosphorylation of retinoblastoma protein (pRB), which controls the G1 to S phase transition by inhibiting the E2F transcription factors, thereby causing cell cycle arrest [17]. p21 and p16 are the most commonly used biomarkers of cellular senescence [18]. Epigenetic changes, which include DNA methylation, histone modification, chromatin remodeling, and transcription of non-coding RNAs, develop during DDR and also contribute to cellular senescence through the regulation of p53 expression [19]. Chromatin structure becomes more compact during aging due to epigenetic mechanisms. Depletion of nuclear lamina protein lamin B1 (LMNB1) leads to the formation of senescence-associated heterochromatin foci (SAHF), which is a transcriptionally inactive form of chromatin and contributes to the maintenance of aging processes in cells [20].

### 2.2. Lysosome Dysfunction

The most widely used lysosomal marker of senescent cells is a specific form of β-galactosidase, Sa-β-gal. High levels of SA-β-gal have been found both in cellular models of aging and in the tissues of old animals and humans [21]. However, the question arises of whether high SA-β-gal activity is truly an indicator of cellular aging rather than a surrogate marker for high lysosomal content or activity. In the study of doxorubicin-induced senescence of neurons, high levels of SA-β-gal activity have been shown in the hippocampus of 24-month-old as well as 3-month-old mice [22]. This means that high SA-β-gal activity is not the mechanism of cellular senescence and can be considered only as a biochemical marker, since inhibition of the enzyme is not able to prevent aging.

The increase in lysosome content in senescent cells occurs as a result of the cellular response to the accumulation of damage products or due to the arrest of cell division [23]. One of the signs of cellular senescence associated with dysfunction of lysosomes is the accumulation of lipofuscin, intralysosomal autofluorescent non-degradable complex containing oxidation products of polyunsaturated fatty acids and metals, formed as a result of oxidative stress and disruption of the balance between damaged proteins and their proteolysis in the cell. Accumulation of lipofuscin pigment interferes with the activity of lysosomal enzymes, which leads to the progression of lysosomal dysfunction and autophagy disorders [24].

Decreased autophagy is one of the main mechanisms of cellular senescence and contributes to organelle dysfunction, accumulation of protein aggregates, decreased pathogen elimination, and increased inflammation [25]. However, there are also controversies regarding the mechanisms of aging in the context of autophagy, as a number of studies have shown a relationship between autophagy activation and cellular senescence. For example, an increase in autophagy associated with age was demonstrated in hematopoietic stem cells, enhancing their regenerative capacity, but the mechanisms stimulating autophagy and maintaining the functionality of senescent hematopoietic stem cells are unclear [26]. Currently, the possibility of using biologically active substances as autophagy inducers is being widely studied, since it is a promising strategy in the prevention of senescence [27].

### 2.3. Mitochondria Dysfunction

Mitochondria are the powerhouses of the cell, responsible for producing adenosine triphosphate (ATP) through oxidative phosphorylation playing crucial roles in regulating metabolic pathways, calcium balance, apoptosis, and ROS production [28]. Mitochondrial dysfunction in senescent cells is an important area of research in the fields of aging and cellular biology. Mitochondrial dysfunction in senescent cells is characterized by increased ROS production and reduced ATP production, so energy deficit and damage of cellular components due to oxidative stress can impair cellular functions [29]. Moreover, senescent cells may shift towards glycolysis for energy production because of altered mitochondrial respiration, leading to metabolic dysregulation. The reduction of mitochondrial respiration was demonstrated in cellular models of doxorubicin-induced and replicative senescence of human vascular smooth muscle cells using the Agilent Seahorse XF Cell Mito Stress by 36% and 78%, respectively. In the same study, ATP production by mitochondria decreased by 34% in doxorubicin-treated cells and by 76% in old cells passaged 15–17 times [30].

Oxidative stress theory is considered as one of the key pathogenetic mechanisms of aging. Oxidative stress also plays a crucial role in the development of age-related diseases [31]. The fact that mitochondria are the main source of ROS in the cell allows them to be considered as a central signaling center that regulates cellular aging [32]. In particular, the consequences of oxidative stress due to dysfunctional mitochondria damage telomeres, promoting cellular aging. A number of studies have also shown the inhibitory effects of oxidative damage on telomerase activity [33,34]. In a model of mice deficient in superoxide dismutase (SOD), a mitochondrial enzyme that provides antioxidant protection of the organism by protecting cells from the effects of ROS, higher levels of double-stranded DNA breaks were shown in the kidney tissue of Sod1-/- mice in comparison wild type mice, along with increased expression of cell cycle arrest markers p16 and p21, increased numbers of Sa-β-gal+ cells, and higher levels of SASP factors IL-6 and IL-1β [35]. Moreover, ROS affect not only cellular organoids but also induce mitochondrial DNA (mtDNA) strand breaks, causing mutations in mtDNA with significant disruption to DNA replication and transcription processes as well as activation of signaling pathways such as the p53 pathway, which plays a role in regulating the cell cycle and apoptosis. The persistent impact of mtDNA mutations on energy metabolism can be considered as a central driver of cellular senescence [36].

The key mechanisms of mitochondrial dysfunction in the pathogenesis of cellular senescence are disturbances in mitophagy and mitochondrial biogenesis, as well as impaired mitochondrial dynamics; that is, the balance of mitochondrial division and fusion [37]. The combination of these processes has been identified as a possible mechanism of mitochondrial quality control (MQC) [38]. Autophagy is an important aspect of MQC, since the functions of mitochondria and lysosomes are interconnected in the pathogenesis of aging. A decrease in the oxidative capacity of mitochondria causes activation of the lysosomal transcription factor EB, which enhances the synthesis of lysosomal proteins and induces autophagy. It has been shown that lipofuscin granules are localized together with depolarized mitochondria in the myocytes of old rodents and humans, confirming the fact that the mitophagy–lysosome system is a key regulator of mitochondrial quality control [39]. Senescent cells are also protected from both internal and external pro-apoptotic signals due to the activation of senescent cell anti-apoptotic pathways (SCAP) and, accordingly, increased expression of anti-apoptotic proteins of the BCL-2 family, which control the permeability of the outer mitochondrial membrane and the release of pro-apoptotic molecules. An increase in mitochondrial content during aging has been shown in various types of cells including fibroblasts, mesenchymal stem cells, hepatocytes, and pancreatic β-cells [40].

Recent evidence suggests that mitochondrial microRNAs (mitoRNAs), derived from the nuclear or mitochondrial genome, play a key role in controlling mitochondrial function and metabolism. For example, mitoRNAs such as miRNA-146a and miRNA-34a, which affect ROS production and susceptibility to apoptosis, have been shown to be increased in endothelial cells during replicative senescence. Expression of miRNA-21 is increased in both replicative senescence and SIPS, resulting in decreased mitochondrial fatty acid oxidation and mitochondrial respiration, thereby shifting cellular metabolism toward glycolysis [41]. Despite active research, miRNAs remain a relatively unexplored area of mitochondrial biology, which opens up prospects for future research to create new diagnostic and therapeutic approaches.

### 2.4. Senescence-Associated Secretory Phenotype

Senescent cells secrete a large number of biologically active substances called the senescence-associated secretory phenotype (SASP). The most well-known factors of SASP include cytokines, interleukin (IL)-6, IL-8, IL-1β, and IL-17a, chemokines, MCP-1–4, MIP-1α (MIP-3α), GROα (and γ), matrix proteases and their inhibitors (MMP 1, 3, 9, and 12 and TIMPs), growth factors, TGF-β and VEGF, and adhesion molecules, ICAM-1 [42]. SASP develops as a result of activation of the SASP pathways due to DDR in senescent cells, as well as oxidative stress due to dysfunction of mitochondria and impaired autophagy [43]. The significance of SASP is controversial. On the one hand, SASP is a pro-inflammatory secretome affecting actively functioning cells, supporting aging. In addition, there is growing evidence that senescent cells may contribute to tumor progression. On the other hand, the secretion of SASP factors stimulates chemotaxis of immune cells responsible for antitumor protection, elimination of senescent cells, and, ultimately, repair of damaged tissues [44].

Transcription of SASP factors is regulated by the nuclear factor-κB (NF-κB) and p38-MAPK pathways in response to aging stimuli, in particular, reactive oxygen species (ROS) [45]. Activation of NF-κB in senescent cells is associated with DNA damage signaling, cGAS-STING, and p38/MAPK pathways. NF-κB is a main regulator of SASP, influencing the expression of target genes, which leads to the progression of senescence. This fact is supported by a number of studies showing that inhibition of the NF-κB pathway in senescent cells reduces the expression of SASP factors [46]. In contrast, persistent activation of NF-κB leads to chronic secretion of proinflammatory factors, and a continuous feedback loop is established between NF-κB and SASP, maintaining cellular senescence [47].

Protein kinase mTOR, the mechanistic target of rapamycin, is also a regulator of protein synthesis that promotes the secretion of SASP factors for maintaining cellular senescence by activating lysosome biogenesis [48]. Cell surface proteins expressed in senescent cells, in particular, CD36, Toll-like receptors (TLRs), and receptors for advanced glycation end products, participate in the regulation of SASP [49]. The secretion of SASP factors determines the main effects of senescent cells. Cellular senescence helps maintain homeostasis and promotes regeneration and antitumor protection, since DNA damage activates aging in cells leading to restriction of division and preventing cancerogenesis. At the same time, long-term exposure to SASP factors can lead to chronic low-intensity inflammation, which is called inflammaging and is associated with various age-related diseases, including cardiovascular and neurodegenerative disorders [50,51]. Several studies have shown that the inflammatory microenvironment can induce pathological changes with hallmarks of cellular senescence regardless of age. For example, one study found significantly higher presence of senescent markers such as p16, lipofuscin, and β-galactosidase along with excessive secretion of SASP factors, IL-1β, IL-6, IL-8, MMP-1, MMP-3, and TNF-α in patients with periodontitis in all age groups compared to healthy subjects [52]. These data demonstrate a predisposition of cells to increased senescence in inflammatory conditions, which highlights the prospect of cellular senescence as a treatment target in chronic inflammatory diseases. SIPS has distinctive features compared to time-dependent senescence, especially in terms of SASP, since SIPS is characterized by excessive secretion of SASP factors and ROS production [53]. In addition, there is a concept of cellular reprogramming associated with aging that results in the “stemness” of senescent cells, allowing them to avoid cell cycle arrest, allowing these cell subsets to be considered as pluripotent stem cells that have an enhanced ability to initiate tumor development. For example, p53-induced senescence in acute lymphoblastic leukemia and acute myeloid leukemia models has been shown to reprogram non-stem leukemia cells into leukemia-inducing stem cells [54]. Thus, studies aimed at investigating the role of SASP in the pathogenesis of aging and the development of SASP-based therapeutic interventions should take into account the characteristics of the SASP of senescent cells in terms of the dynamics of the SASP composition, the context of aging, and its multifaceted influence on the microenvironment over time [55].

While some of the most widely studied senescent cell markers such as cell cycle arrest markers, SA-β-gal+ staining, and nuclear changes are applicable for assessing cellular senescence in most organs and tissues, a number of SASP factors are tissue-specific. In addition, it is necessary to consider that SASP factors can be secreted not only by senescent cells but also by non-senescent cells. A recent review characterizes the hallmarks of senescent cells depending on tissue types and highlights priority markers of senescence that are specific for the most cell types; in particular, p16 and p21, γH2AX, loss of lamin B1, SASP factors IL-6, IL-1α, IL-1β, SA-β-gal, and increased expression of BCL-2 family proteins [56]. Table 1 demonstrates the heterogeneity of the most-studied SASP factors and other senescent markers in different organs.

Senescent cell markers may differ depending on the context of cellular senescence; in particular, in aging, cancer, or regeneration [57]. Thus, activation of CDK inhibitors p16 and p21, increased activity of SA-β-gal, accumulation of lipofuscin, nuclear changes, and SASP phenotype are the most specific senescence markers in aging [58]. The p53 protein is one of the key markers of cellular senescence in cancer; however, cancer cells are heterogeneous, and only a part of them may be positive for senescence markers [59]. In addition, an altered SASP profile is observed in cancer cells, which not only contributes to tumor suppression through aging of cancer cells but also triggers cells of the microenvironment, leading to tumor progression and metastasis [60]. During the regeneration process, cellular senescence is a temporary mechanism aimed at eliminating damaged cells and is mainly mediated by SASP factors, while the expression of cell cycle arrest markers such as p16 and p21 predicts a longer wound healing time [61].

## 3. Implications for Anti-Aging Therapy

**Figure 1:**
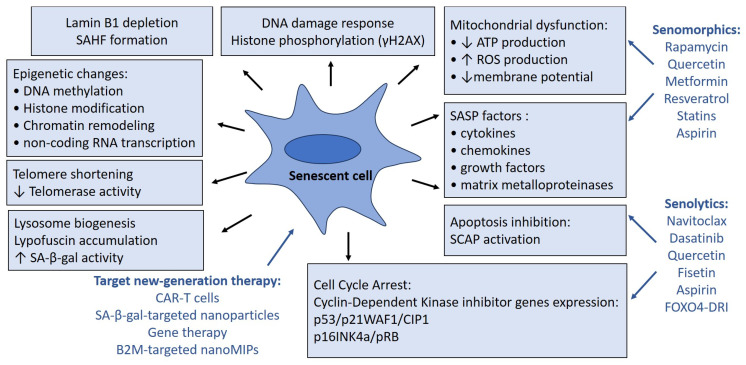
Therapeutic interventions targeting cellular senescence.

Since it is well-known that the accumulation of senescent cells is associated with aging and the development of age-associated diseases, targeting of senescent cells is now considered as the most promising strategy for longlife intervention [62]. Figure 1 demonstrates the hallmarks of aging cells and therapeutic interventions targeting cellular senescence. Geroprotective preparations are represented by small-molecule compounds exhibiting cytotoxicity toward senescent cells (senolytics) and therapeutics inhibiting oxidative stress and harmful effects of SASP (senomorphics). Novel anti-aging approaches include immunotherapy directed at surface antigens specifically upregulated in senescent cells; in particular, chimeric antigen receptor (CAR) therapies and senolytic vaccines [63].

γH2AX, phosphorylated form of histone H2AX; ATP, adenosine triphosphate; B2M, macroglobulin-β2; CAR-T, chimeric antigen receptor of T cells; FOXO4-DRI, FOXO4-D-Retro-Inverso; IL, interleukin; MCP-1, monocyte chemoattractant protein-1; ROS, reactive oxygen species; SASP, senescence-associated secretory phenotype; SA-β-gal, senescence-associated β-galactosidase; SAHF, senescence-associated heterochromatin foci; SCAP, senescent cell anti-apoptotic pathways; TGFβ, transforming growth factor β.

### 3.1. Senolytic Therapy

The first generation of senolytic preparations was developed based on the hypothesis of SCAP activation in senescent cells, which causes resistance to apoptosis in to 70% of senescent cells. The best-known representatives of this class of drugs are dasatinib, quercetin, and fisetin, which in mono- or complex therapy have been studied not only in preclinical trials but also in phase 1 and 2 clinical trials, demonstrating safety and efficacy [64]. The combination of preparations of dasatinib and quercetin is the earliest senolytic strategy and the most studied so far. Dasatinib is a tyrosine kinase inhibitor initially used to treat chronic myeloid leukemia, acute lymphoblastic leukemia, and a number of other oncological diseases [65]. It was one of the first drugs targeting antiapoptotic pathways that was studied as a senolytic agent. It was shown in experimental models of aging induced by ionizing radiation that dasatinib suppressed the viability of SA-β-gal+ senescent human preadipocytes and mouse embryonic fibroblasts, but it was ineffective against human umbilical vein cells and senescent bone marrow mesenchymal stromal cells. Therefore, the senolytic activity of dasatinib was further studied in combination with another anti-aging agent of natural origin, quercetin, which is able to act synergistically, increasing therapeutic efficacy while minimizing side effects. The anti-aging effect of quercetin is explained by the regulation of signaling pathways, leading to the induction of apoptosis in senescent cells by activating p53 and suppressing the genes of the BCL-2 proteins, as well as to the reduction of oxidative stress [66]. The senolytic effect of the combination of dasatinib and quercetin affected cellular senescence in a wider range of senescent cell types [67,68] and was confirmed in animal models demonstrating that treatment with dasatinib and quercetin resulted in significant decrease in Sa-β-gal+ cells and expression of cell cycle inhibitors p16 and p21 in different tissues of old or doxorubicin-treated C57BL/6 mice [69,70,71]. The senolytic efficacy of the combination of dasatinib and quercetin was demonstrated in a pilot clinical trial in patients with diabetic kidney disease. The study revealed that 3 days of administration of a combination of dasatinib and quercetin resulted in a reduction in circulating levels of SASP factors, IL-1β, IL-6, MMP-9, 12; decreased p16 and p21 expression in skin epidermis and adipose tissue; and reduction in SA-β-gal+ adipocytes in adipose tissue [72]. The senolytic activity of the combination of dasatinib and quercetin was evaluated in clinical studies in postmenopausal women, in patients with Alzheimer’s disease, and in patients with idiopathic pulmonary fibrosis [73,74,75].

Fisetin is another natural preparation that exerts a number of senolytic effects targeting anti-apoptotic pathways in senescent cells. When evaluating the senolytic efficacy of a panel of 10 polyphenols in a model of induced aging of mouse and human fibroblasts, it was shown that fisetin possessed the most prominent effects in terms of senescence markers such as the number of SA-β-gal+ cells, SCAP activation, p16 and p21 expression, and SASP factor secretion [76]. Another study confirmed the senolytic effect of fisetin in a cellular model of replicative senescence of human dermal fibroblasts in terms of SA-β-gal+ cells numbers [77]. A study in old sheep showed that fisetin administration reduced the number of SA-β-gal+ neurons, astrocytes, and microglial cells in the cerebral hemispheres and hippocampus [78]. Fisetin was also shown to inhibit ROS-induced senescence of vascular smooth muscle cells regulated by the PTEN-PKCδ-NOX1-ROS signaling pathway [79]. The efficacy of fisetin is currently being widely studied in a number of clinical trials in patients with age-associated diseases, such as osteoarthritis, frailty, type 2 diabetes mellitus, and chronic kidney disease, as well as COVID-19 (SARS-CoV-2) infection in the elderly [80].

Navitoclax (ABT263) specifically inhibits anti-apoptotic proteins of the BCL family, thereby activating the caspase signaling pathway of apoptosis [81]. Navitoclax has been shown to induce apoptosis of senescent cells in human umbilical vein endothelial cells (HUVECs), human fetal lung fibroblasts (IMR-90), and mouse embryonic fibroblast (MEF) cell lines, cultured mice chondrocytes in a model of osteoarthritis, senescent renal tubular epithelial cells in a mouse model of chronic kidney disease, and UV-irradiated senescent melanocytes [81,82,83]. Navitoclax also reduced the number of senescent brain endothelial cells in a model of accelerated aging in mice [84]. Navitoclax is used in the treatment of some kinds of cancer in combination with other chemotherapeutic preparations; however, it has serious side effects such as thrombocytopenia and neutropenia. Therefore, its clinical use as a senolytic agent requires further study [85]. Table 2 demonstrates molecular targets and effects of senolytics.

Recent advances in senolytic therapy include new preparations effectively targeting senescent cells. In particular, heat shock protein 90 (HSP90) inhibitors promote activation of tumor suppressor protein p53 affecting apoptosis and DNA repair. It has been shown that inhibition of HSP90 inhibitor reduces SA-β-gal activity and p16 expression in senescent MEF [93]. Transcriptional factor fork head box O 4 (FOXO4) interacts with the proapoptotic protein p53, inhibiting apoptosis of aging cells [94]. FOXO4-D-Retro-Inverso (FOXO4-DRI) is a peptide-based preparation that induces apoptosis through p53 activation due to inhibition of the interaction between proteins FOXO4 and p53. The ability of FOXO4-DRI to induce apoptosis and reduce the viability of senescent cells compared to control cells has been shown in in cigarette smoke-induced senescent lung fibroblasts and in the IMR90 cell line [95,96].

The identification of senescence-associated antigens, cell surface molecules on senescent cells, has pointed to another promising direction for developing new diagnostic and treatment approaches. Senolytic vaccines are developing based on chimeric antigen receptor of T cells (CAR-T), which binds to specific cell surface proteins expressed in senescent cells; in particular, uPAR. uPAR-specific CAR-T cells have been shown to effectively eliminate senescent cells in vitro and in vivo [97]. The use of CAR-T therapy has proven effective in the treatment of malignancies in hematology and is currently undergoing clinical trials for use in prostate cancer, glioblastoma, and autoimmune diseases [98]. Personalized anti-aging strategies using CAR-T cell preparations are extremely promising but require further study since immune complications are possible, leading to cytokine release syndrome and immune effector cell-associated neurotoxicity syndrome. Additionally, the destruction of senescent cells using this class of preparations is difficult to stop if necessary. Second-generation senolytics were also created based on silica nanoparticles coated with galactooligosaccharides containing chemotherapeutic agents that are activated by Sa-β-gal for target elimination of senescent cells, which was confirmed in cellular and animal models [99]. Another class of molecularly imprinted nanoparticles (nanoMIPs) recognizes an epitope senescent marker microglobulin β2 (B2M) that allows for detecting and selectively killing aging cells [100]. Targeted delivery of senolytics using cargo nanoparticles can significantly reduce the toxicity of senolytics to healthy cells. Finally, anti-aging gene therapy allows for the elimination of cells expressing senescence-associated proteins such as p16, which significantly reduces the effects of therapy on normally functioning cells, thereby increasing the targeting of the effect and therefore the safety and effectiveness of treatment [100]. The anti-aging effectiveness of gene therapy directed at telomerase activation has been proved in animal models. However, the most serious concerns regarding telomerase gene therapy include possible risk of cancer induction, but in mice, the treatment increased longevity without elevation of cancer frequency [101].

### 3.2. Senomorphic Therapy

Another class of geroprotective preparations includes senomorphics, reducing SASP via regulation of the expression of mTOR, NF-κB, ATM, p38-MAPK, JAK/STAT, and other signaling pathways. These preparations indirectly inhibit the development of cellular senescence without eliminating senescent cells [102].

Rapamycin is a macrolide compound isolated from the bacterium Streptomyces hygroscopicus, possessing immunosuppressive properties. The molecular target of rapamycin is the TOR gene and mTOR signaling pathway, serine/threonine protein kinase belonging to the phosphatidylinositol 3-kinase (PI3K) family, which plays a key role in the regulation of energy metabolism. Numerous studies have shown that rapamycin suppresses induced cellular senescence and the production of SASP factors in various murine and human cell lines, as well as in animal models of aging. Rapamycin has been also shown to increase the lifespan of yeast, worms, flies, and mice [103]. Experiments in animal aging models have demonstrated that rapamycin treatment delays cataract development, reduces age-related muscle loss, and promotes periodontal bone regeneration [104,105]. It has been demonstrated that rapamycin improves clinical and laboratory parameters in cardiovascular, oncological, and neurodegenerative diseases, but it can result in a number of side effects such as immunosuppression, hyperglycemia, and hyperlipidemia. However, the level of toxicity of rapamycin for humans is relatively low, so rapamycin is considered as a very promising geroprotective agent [103,106].

Metformin is the most widely used first-line preparation for the treatment of type 2 diabetes mellitus. Metformin is a pleiotropic senomorphic agent that affects several molecular targets, including the mTOR, NF-κB, and JAK/STAT pathways [107]. Numerous studies have shown the efficacy of metformin on cellular aging parameters such as secretion of SASP factors, mitochondrial dysfunction, telomere shortening, and epigenetic changes; in clinical trials, metformin reduced the progression of various age-associated chronic diseases [108]. It was shown that metformin regulates the metabolism of HEK293T cells through the lysosomal pathway and extends the lifespan of C. elegans and mice [108]. Long-term administration (3 years) of metformin has tissue- and cell-specific geroprotective effects in male cynomolgus monkeys, neuroprotective effects, positive changes in aging biomarkers, and a tendency towards rejuvenation of the multidimensional aging clock after treatment [109]. However, large-scale, double-blinded, randomized, placebo-controlled studies are required to develop personalized geroprotective strategies given limitations including the selective efficacy and low bioavailability of metformin.

Other classes of preparations possessing anti-inflammatory, anti-oxidant, and anti-cytokine properties may be considered as senotherapeutic agents. In particular, resveratrol and other polyphenols such as kaempferol, apigenin, genistein, and others exert senomorphic effects through the suppression of signaling pathways associated with oxidative stress, SIRT1 activation, and stimulation of autophagy [110]. It was demonstrated that the hydroxy-methyl-glutaryl-coenzyme A (HMG-CoA) reductase inhibitor simvastatin reduces the secretion of SASP factors and ROS production and improves mitochondrial respiration in cellular models of doxorubicin-induced and replicative senescence of VSMCs [30]. Table 3 demonstrates molecular targets and effects of senomorphics.

Despite the active participation of bioactive substances of SASP in the pathogenesis of aging, isolated suppression of the secretion of SASP factors cannot be considered as a strategy for geroprotective therapy. Therefore, other mechanisms of aging must also be taken into account. For example, it has been shown that taking a non-steroidal anti-inflammatory preparation aspirin, which effectively suppresses SASP, leads to a decrease in telomerase activity, and accordingly, telomere length in patients with diabetes mellitus [112]. However, another study showed that aspirin suppresses the expression of p53 and p21 in a model of doxorubicin-induced senescence of human fibroblasts and in mouse embryos [113].

### 3.3. Clinical Trials of Anti-Aging Agents

The anti-aging efficacy of some geroprotective agents in cellular and animal models allowed for the development of clinical trials of these preparations in patients with various age-associated conditions and diseases. Table 4 demonstrates the results of clinical trials on the effects of geroprotective preparations in age-associated conditions and diseases.

Preparations effective against cellular senescence in experimental models are in clinical trials and still need to be thoroughly evaluated for their efficacy and safety as anti-aging agents. Second-generation targeted senotherapeutics are in early stages of development, and further studies are needed to assess their geroprotective potential.

## 4. Conclusions

Cellular senescence underlies aging and the development of age-associated diseases. However, when developing approaches to senolytic therapy, it is especially important to remember the physiological functions of cellular senescence. Cellular aging is a particularly complex concern in the context of tumor development, since it simultaneously serves as a mechanism for suppression and progression of carcinogenesis due to the secretion of SASP factors [118]. Thus, senescent cells exhibit considerable heterogeneity, which complicates the development and implementation of geroprotective therapy. The hallmarks of senescent cells depend on tissue type and the phenotype of senescent cells. However, among the variety of bioactive substances, signaling pathways, and structural rearrangements associated with cellular aging, it is difficult to identify a universal marker of senescent cells. Given the complexity of detecting senescent cells, further studies should be conducted to reveal features of cellular aging using modern methods based on omics technologies with bioinformatics data analysis to develop relevant models for the assessment of cellular senescence. Current research is also exploring the mechanisms underlying mitochondrial dysfunction in senescent cells, since targeting mitochondria is a potential strategy for developing senolytic therapy to promote healthy aging. Currently, multiple clinical studies are devoted to investigating the senolytic and senomorphic activity of existing preparations; novel geroprotective strategies are aimed at target elimination of senescent cells and include preparations based on nanoparticles coated with galactooligosaccharides, activated by Sa-β-gal, B2M-targeted nanoMIPs, T cell vaccines binding to specific cell surface proteins expressed in senescent cells, and gene therapy. However, these approaches are undergoing early stages of clinical trials and need further investigation to evaluate their efficacy and safety as anti-aging agents.

## Figures and Tables

**Table 1:** The most-studied SASP factors and other senescent markers in organs and tissues.

Organs and Tissues	SASP Factors	Other Senescent Markers
Adipose tissue	IL-6, TNF-α, IL-1β, IL-1α, MCP-1, IL-8, MMP 3, 12	p16, p21, p53, γH2AX, SA-β-gal, LMNB1
Skin	IL-6, TNF-α, IL-1β, IL-1α, TGF-β, MCP-1, IL-8, MMP 1, 3, 9	p16, p21, γH2AX, SA-β-gal, lipofuscin, LMNB1, telomere length
Cardiovascular system	IL-6, TNF-α, IL-1β, IL-1α, TGF-β, MCP-1, MMP 3, 9, 12	p16, p21, p53, γH2AX, SA-β-gal, telomere length
Bone marrow	IL-6, TNF-α, IL-1β, IL-1α, TGF-β, MCP-1, MMP 9, 12, ICAM-1, IL-17a, IFN-γ, VEGF	p16, p21, p53, SA-β-gal
Central nervous system	IL-6, TNF-α, IL-1β, IL-1α, TGF-β, MCP-1, IL-8, MMP 3, 12, TIMP	p16, p21, p53, γH2AX, SA-β-gal, LMNB1, BCL-2
Kidneys	IL-6, TNF-α, IL-1β, TGF-β, MCP-1, MMP 1, 12	p16, p21, γH2AX, SA-β-gal, telomere length
Liver	IL-6, TNF-α, IL-1α, TGF-β, MCP-1, MMP 1, 3	p16, p21, p53, γH2AX, SA-β-gal
Lungs	IL-6, TNF-α, IL-1β, IL-1α, IL-8, TGF-β, MMP 12, VEGF	p16, p21, p53, γH2AX, SA-β-gal
Pancreas	IL-6, TNF-α, IL-1β, IL-1α, TGF-β, ICAM-1	p16, p21, p53, γH2AX, SA-β-gal
Ovary	IL-6, IL-1β, IL-1α, TGF-β, MCP-1, IL-8, TIMP	p16, p21, γH2AX, SA-β-gal, lipofuscin, BCL-2
Bone tissue	IL-6, TNF-α, IL-1β, IL-1α, TGF-β, MCP-1, IL-8, MMP 1, 3, 9, 12, ICAM-1, IL-17a, IFN-γ, VEGF, TIMP	p16, p21, p53, LMNB1, BCL-2

γH2AX, phosphorylated form of histone H2AX; IFN-γ, interferon-γ; ICAM-1, intercellular adhesion molecule 1; IL, interleukin; LMNB1, lamin B1; MCP-1, monocyte chemoattractant protein-1; MMP, matrix metalloproteinases; SASP, senescence-associated secretory phenotype; SA-β-gal, senescence-associated β-galactosidase; TIMP, tissue inhibitor of metalloproteinases; TGF-β, transforming growth factor β, VEGF, vascular endothelial growth factor.

**Table 2:** Molecular targets and effects of senolytics.

Senolytic Molecule	Molecular Targets	Effects
Dasatinib	Primary target: SCAP inhibition (tyrosine kinases, ephrin receptors) [65,86]	↓ SA-β-gal+ cells in models of induced senescence of BM-MSC, adipocyte progenitors, human endothelial cells, human gingival keratinocytes, in skeletal myocytes of old C57BL/6 mice, and in ovarian cells of doxorubicin-treated C57BL/6 mice [67,68,69,70]. ↓ expression of cell cycle inhibitors p16 and p21 in jejunum epithelial cell of old C57BL/6 mice and in ovarian cells of doxorubicin-treated C57BL/6 mice [70,71].
Quercetin	SCAP inhibition (PI3K/AKT, BCL-2/BCL-xL, MDM2, TP53/P21) [66,86] MAPK pathway inhibition [87] Cyclooxygenase inhibition [87] Nrf2/HO1 activation [88] SIRT1 activation [89]	
Fisetin	NF-κB and PTEN-PKCδ-NOX1 pathway downregulation [79] Nrf2 pathways activation [90] MAPK pathway inhibition [91] PI3K/AKT pathway activation [91] BCL-2 protein family inhibition [92] SIRT1 activation [89]	↓ expression of cell cycle inhibitors p16 and p21 in ovarian cells of doxorubicin-treated C57BL/6 mice [70]. ↓ SA-β-gal+ cells in murine and human fibroblasts, astrocytes, microglial cells in old sheep [76,77,78].
Navitoclax	BCL-2 protein family inhibition [81]	↓ number of senescent bone marrow hematopoietic stem cells and myoblasts in mice; in HUVECs, IMR-90 and MEF cell lines; in UV-irradiated senescent melanocytes; in brain endothelial cells in a model of accelerated aging in mice [81,82,83,84].

BM-MSC, bone marrow-derived mesenchymal stem cells; HUVEC, human umbilical vein endothelial cells; IMR-90, human fetal lung fibroblasts; MAPK, mitogen-activated protein kinase; MEF, mouse embryonic fibroblasts; NF-κB, nuclear factor-κB; Nrf2, nuclear factor related factor 2; SA-β-gal, senescence-associated β-galactosidase; SCAP, senescent cell anti-apoptotic pathways; SIRT1, sirtuin 1.

**Table 3:** Molecular targets and effects of senomorphics.

Senomorphic Molecule	Molecular Targets	Effects
Rapamycin	Primary target: mTOR pathway [103]	↑ lifespan in mice; ↓ cataract development, ↓ age-related muscle loss, ↑ periodontal bone regeneration [103,104,105].
Quercetin	NF-κB, JNK, ERK, JAK-STAT, mTOR pathway downregulation [111]	↓ SASP and SA-β-gal activity [67].
Metformin	AMPK-dependent pathways, NF-κB, JAK-STAT, mTOR pathway downregulation [107]	↑ lifespan of C. elegans and mice [108]; restoration of tissue metabolism and improvement of clinical parameters in patients with age-associated disorders including diabetes mellitus, cardiovascular diseases, neurodegenerative diseases, degenerative musculoskeletal diseases, obesity [108]; ↓ senescence biomarkers in monkeys, neuroprotective effect, tendency to rejuvenation of multidimensional aging clock [109].
Resveratrol	SIRT1 activation [110] NF-κB pathway inhibition [110]	↓ SASP and ROS production [110].
Simvastatin	HMG-CoA reductase inhibition [30]	↓ SASP and ROS production, ↑ mitochondrial respiration in aging cells [30].

AMPK, adenosine monophosphate-activated kinase; HMG-CoA, hydroxy-methyl-glutaryl-coenzyme A; mTOR, mechanistic target of rapamycin; NF-κB, nuclear factor-κB; SA-β-gal, senescence-associated β-galactosidase; SASP, senescence-associated secretory phenotype; SIRT1, sirtuin 1; ROS, reactive oxygen species.

**Table 4:** Clinical trials of geroprotective agents for age-associated conditions.

Geroprotective Agents	Clinical Trial	Age-Associated Conditions	Study Results
Rapamycin	NCT03103893	Dermal thickness and senescence	Clinical improvement in skin appearance, improvement in histological appearance of skin tissue, histological markers of aging, increase in collagen VII [114] (phase II)
	NCT05414292	Muscle mass during physical training in healthy individuals aged 50–90 years	N/A (recruiting healthy male volunteers)
	NCT04200911	Cognitive functions in early Alzheimer’s disease	N/A (early phase I)
Dasatinib + Quercetin	NCT02848131	Chronic kidney disease	Reduction in SASP, p16, and p21 expression in patients with diabetic kidney disease in combination with Dasatanib [72] (phase II)
Dasatinib + Quercetin + Fisetin	NCT04313634	Bone resorption/bone formation markers in elderly women	Reduction in bone resorption in postmenopausal women in combination with Quercetin and Fisetin [75,115] (phase II)
Fisetin	NCT04210986	Osteoarthritis-related articular cartilage degeneration	N/A (phase II)
	NCT03325322	Chronic kidney disease	N/A (phase I)
Navitoclax	NCT02079740	Advanced or metastatic solid tumors	MAPK pathway inhibition, reductions in KRAS/NRAS mutation levels [116] (phase II)
	NCT03181126	Relapsed/refractory acute lymphoblastic leukemia or relapsed/refractory lymphoblastic lymphoma	Complete remission (60% patients) [117]
	NCT06156774	Sarcopenia and simplified geriatric assessment in lymphoma patients	N/A (observational study)
CAR-T cell therapy	NCT04300998	Older patients with hematologic malignancies	N/A (observational study)

CAR-T cell, chimeric antigen receptor of T cells; MAPK, mitogen-activated protein kinase; N/A, not available; SASP, senescence-associated secretory phenotype.

## Data Availability

Not applicable.
